# An investigation into patterns of Alcohol drinking in Scotland after the introduction of minimum unit pricing

**DOI:** 10.1371/journal.pone.0308218

**Published:** 2024-08-01

**Authors:** Duyen Thuy Nguyen, Michael Donnelly, Minh Van Hoang, Ciaran O’Neill

**Affiliations:** 1 Center for Public Health, School of Medicine, Dentistry and Biomedical Sciences, Queen’s University Belfast, Belfast, United Kingdom; 2 Center for Population Health Sciences, Hanoi University of Public Health, North Tu Liem district, Hanoi, Vietnam; Cardiff University, UNITED KINGDOM OF GREAT BRITAIN AND NORTHERN IRELAND

## Abstract

**Background:**

In 2018, Scotland became the second country to implement minimum unit pricing (MUP) for all types of alcoholic beverages. The aim of this study was to examine the effect of the policy.

**Method:**

Three national household-level surveys were used: Scottish Health Surveys (2008–2021), Health Surveys in England (2011–2019), and Northern Ireland Continuous Household Survey (2011–2015). First, a generalized ordered logistic model examined patterns of drinking solely in Scotland from 2008–2021 covering current drinking, drinking categories and the weekly consumption (in alcohol units). Secondly, difference-in-difference (DID) analysis was employed to examine changes in “social drinking” behaviours in Scotland after the announcement in 2012 (2011–2015, Northern Ireland and England as comparators) and after the adoption of the policy in 2018 (England as a comparator, with two timeframes 2016–2019 and 2013–2019).

**Results:**

Overall, drinking in Scotland began to decline prior to 2012 and dropped further with the enactment of MUP in 2018. In response to MUP, the likelihood of abstention increased along with a slight decrease in the prevalence of heavy drinking. The overall amount of drinking fell by about 8% after 2012 and 12% after 2018 (as compared to 2008–2011 level), with a significant decline seen in moderate drinkers but not of those who drank at hazardous or harmful levels. The DID analyses confirmed the reduction in current drinking in Scotland starting since 2012 and continued post-MUP in 2018.

**Conclusion:**

This study points to the impact of MUP in Scotland with a potential role for ‘policy signalling’ by the Scottish Government’s with a multiple-buy discount ban and MUP’s announcement since 2011–2012. Indications of impact include a clear decline in alcohol consumption levels and a small but noteworthy change in prevalence of overall drinking and heavy drinking.

## 1. Background

Harmful drinking has long been a source of concern among public health officials in the UK and Ireland [1–3]. The Scottish government has taken a number of additional specific actions beyond standard fiscal and education measures since 2010 to progress alcohol policies including the announcement in 2012 and subsequent adoption in 2018 of minimum unit pricing (MUP) on alcohol [4]. Particular attention was given to the MUP policy as a novel intervention for reducing alcohol consumption given Scotland was one of the first countries to announce a MUP on all types of alcoholic beverages in 2012 and the second jurisdiction to adopt such policy by 2018 (Armenia introduced MUP in 2016) [5,6]. The principle of MUP is to impose a floor price (£0.50 per unit) below which alcoholic products cannot be sold [7]. This regulation directly targets cheaper and higher-strength alcohol products and attempts to ensure that alcohol is not sold at “discount prices” to which users may switch when alcohol becomes more expensive. The aim of the study was to examine the nature and degree of any changes in drinking behaviour in the Scottish adult population and to assess changes induced by MUP after adjusting for secular trends. The study focused on two milestones: the announcement of the policy in 2012 and its adoption in 2018. The first milestone took into account two policy actions directly aimed at curbing alcohol purchase: a ban on quantity discounts for alcohol in 2011 [8] and an announcement of MUP in June 2012 (which at that time was expected to be implemented shortly after [9]). The second milestone focused on the eventual enactment of MUP six years after its announcement due to repeated legal challenges from Scotch Whisky Association [9].

## 2. Method

### 2.1. Data sources

Following the implementation of MUP, early evidence pointed to the impact of MUP in Scotland regarding alcohol consumption and related behaviour from three main data sources [6] including alcohol sales data [10–14]; large sample individual- or household- based survey data commercially collected by market research companies [15–18] and survey-based analyses using smaller samples [6,19,20]. Given the limitation due to commercial sensitivities in previous studies, this study used three governmental-run population-based health surveys representing three constituent parts of the UK: Scotland, England and Northern Ireland to examine the impact of MUP. The examination of MUP policy in Scotland was undertaken in two parts. The first analysis solely focused on analysing drinking patterns in Scotland. The second part employed a difference-in-difference (DID) approach to compare Scotland with two neighbouring jurisdictions—England and Northern Ireland–where distinct policy environments operated.

For Scotland, data were taken from the Scottish Health Survey (SHeS) providing a series of cross-sectional data from 2008–2021 (excluding 2020 data collected by telephone survey due to COVID restrictions). For England, the Health Survey for England (HSE) was employed given its similarities on survey flow and questionnaire structure to SHeS providing a series of cross-sectional samples for the period 2011–2019. For Northern Ireland, Northern Ireland Statistics and Research Agency (NISRA) provided the Continuous Household Survey (NI CHS)–another cross-sectional survey with data available from 2011–2015 (details on Northern Ireland data can be found elsewhere [21]). Datasets for Scotland and England were obtained from the UK Data Archive in June 2023 and data for Northern Ireland CHS was deposited from Northern Ireland Statistics and Research Agency in May 2022. Further details are available in the surveys’ technical reports [22–25]. As the study used anonymised secondary data, it was deemed exempt from ethics approval by Faculty of Medicine, Health and Life Sciences Research Ethics Committee, Queen’s University Belfast (ref no. RGE 23_04).

### 2.2. Statistical analysis strategy

#### 2.2.1. Changes in drinking rate & drinking amount in Scotland

In the first analysis focused on Scotland only, multivariate models were conducted to assess two patterns of drinking: drinking categories and drinking amount across three periods: 2008–2012, 2013–2017, 2018–2021 to test for changes in drinking behaviours after the announcement (2012) and after the enactment (2018) of MUP respectively. To account for the potential impact of COVID-19 on drinking, an additional analysis for Scotland data was also conducted by excluding the last survey wave in 2021 to re-examine the results from the main analysis covering the period from 2008 until 2021 (see S4 File).

For the drinking categories, a generalized ordered logistic model was employed to examine the likelihood of current drinking (abstainers vs. current drinking at any level); heavy drinking (abstainer/ moderate drinking vs. hazardous/ harmful drinking) and; harmful drinking (abstainer/ moderate/ hazardous drinking vs. harmful drinking) across three periods [26]. This model was chosen as it is less restrictive than the ordinal regression model (requires proportional odds assumption) but more parsimonious than multinomial logistic regression [26]. Moderate, hazardous, and harmful drinking were defined using the UK guideline before 2015 in which *moderate drinking* is equivalent to less than 21 units for men and 14 for women per week; *hazardous drinking* if over 21–50 units for men and over 14–35 for women; and *harmful drinking* refers to over 50 units for man and over 35 for women. Occasionally, the term heavy drinking/ drinkers is used to refer to those who drank at a hazardous and harmful level. Current abstainers referred to those reported never drinking or drinking only very occasionally.

For the drinking amount, negative binomial regression models were estimated using the derived weekly alcohol consumption for the overall sample and then for specific groups of moderate drinkers, hazardous drinkers, and harmful drinkers. The total alcohol consumption per week was derived in alcohol units from the self-reported frequency and consumption amounts of beer, spirits, sherry, wine, and alcohol pops over the last 12 months.

All models were multivariate models and included sex, age, ethnicity, marital status, education, employment, smoking, longstanding illness, income, and area deprivation as covariates. These are commonly used as predictors of lifestyle behaviours [27]. A brief description of included variables is provided in S1 File. Since sample weights were available for Scotland and England but not for Northern Ireland, the weighted analyses were performed and specified wherever applicable along with unweighted results.

#### 2.2.2. Difference-in-difference analyses

The DID analyses investigated the outcome of current drinking as binary outcome (1 = currently drinking, 0 = Never/ Very occasionally drink) firstly using a 2x2 DID design and secondly a propensity score matching DID (PSM-DID) design.

To examine the change of drinking following the announcement of MUP in 2012, two DID models were conducted to compare the drinking rate in Scotland vs Northern Ireland (model 1) and in Scotland vs England (model 2). Both models investigated the likelihood of current drinking between two periods: 2011–2012 and 2013–2015.

To examine the change of drinking after the enactment of MUP in 2018, two further DID models were estimated with Scotland and England data but in two different baseline periods. Model 3 examined current drinking in the periods 2016–2017 versus 2018–2019 (consecutive with the timeframe of model 2); while model 4 examined the period from 2013–2017 (from announcement to enactment) and 2018–2019 (after enactment).

The 2x2 DID models took the form of a linear probability model that included the DID interaction term as: $Y=\alpha +\beta_{1}\;Scotland+\beta_{2}\;MUP+\beta_{3}*Scotland*MUP+\omega X+\varepsilon$. Y is the outcome of current drinking. *Scotland* is the variable for treatment group (Scotland = 1) and control group (Northern Ireland or England = 0). *MUP* is the indicator for the year of minimum pricing unit policy in which 0 = before MUP and 1 = after MUP. The interaction term of Scotland and MUP is the DID variable where *β*_3_ represents the effect of MUP policy after accounting for the difference between Scotland and the control group over time. X represents the vector of covariates as listed in the regression analysis for Scotland above.

Next, a matching DID analysis was conducted using Kernel-type Propensity Score Matching (PSM). This analysis was performed due to the violation of parallel trend assumption of DID which is commonly seen in DID study and also the case of this study. Firstly, the propensity scores were generated from the baseline characteristics of the treated group (Scotland) and control group using the set of covariates above known to be related with drinking but not the treatment assignment [28]. The second step was to match and weight using the created propensity scores. The Kernel matching of propensity score matched treated participants (in Scottish sample) to a weighted sum of individuals who have similar propensity scores in the control sample. The weights are inversely proportional to the distance between the treated and control group’s propensity scores. The kernel matching employed the Epanechnikov function with bandwidth of 0.6 as commonly used in the literature [29–32]. Through the matching procedure, a pseudo panel was created in relation to Scotland and a specific, weighted control group (from the Northern Ireland and England sample, respectively) that matched the other group pre-policy. The next step in this DID approach was then performed on the two matched samples, relaxing the parallel trend assumption in drinking at baseline (pre-policy).

## 3. Results

### 3.1. Descriptive statistics

Across three periods, the age structure got older for Scottish population, a higher share of the population obtained higher educational attainment and more people resided in the less deprived areas (see Table 1). No significant changes were observed for the patterns of gender, marital status, employment, and household income. During 2008–2021, there was an increase in population share with longstanding illness but there were fewer people who reported current smoking and current drinking. The rate of hazardous and harmful drinking reduced over time with a slight increase in moderate drinking group. Interestingly, the median amount of weekly alcohol consumption reduced from 4.73 units (2008–2012) to 3.75 units (2013–2017) and 3.67 units (2018–2021). S2 File provides additional comparisons of general characteristics between Scotland versus Northern Ireland and England.

**Table 1:** Descriptive statistics of Scotland from 2008–2021.

Scotland	Weighted (SE)			Unweighted		
	2008–2012	2013–2017	2018–2021	2008–2012	2013–2017	2018–2021
*N*	**33,600**	**22,570**	**14,270**	**33,600**	**22,570**	**14,270**
**Sex**						
Female	52.1% (0.3%)	52.0% (0.3%)	51.8% (0.4%)	56.4%	55.9%	56.8%
Male	47.9% (0.3%)	48.0% (0.3%)	48.2% (0.4%)	43.6%	44.1%	43.2%
**Age groups**						
16–19	6.4% (0.2%)	5.6% (0.3%)	5.4% (0.3%)	3.8%	3.4%	3.1%
20–24	7.5% (0.3%)	7.6% (0.3%)	6.6% (0.4%)	5.0%	5.2%	3.9%
25–34	15.4% (0.3%)	16.0% (0.4%)	16.6% (0.5%)	12.7%	13.1%	11.8%
35–49	26.7% (0.3%)	24.0% (0.4%)	22.7% (0.5%)	26.3%	23.3%	21.7%
50–59	16.2% (0.3%)	17.8% (0.3%)	18.5% (0.4%)	17.2%	18.4%	19.2%
60+	27.8% (0.3%)	29.1% (0.5%)	30.2% (0.6%)	35.1%	36.5%	40.3%
**Marital status**						
Single/ Divorced/ Separated/ Widowed	39.3% (0.4%)	39.5% (0.5%)	39.5% (0.6%)	36.7%	37.6%	37.5%
Married/ cohabited	60.7% (0.4%)	60.5% (0.5%)	60.5% (0.6%)	63.3%	62.4%	62.5%
**Education**						
Lower educated	63.7% (0.4%)	57.4% (0.5%)	49.9% (0.7%)	64.7%	58.5%	48.9%
Higher educated	36.3% (0.4%)	42.6% (0.5%)	50.1% (0.7%)	35.3%	41.5%	51.1%
**Employment**						
Unemployed	44.9% (0.4%)	45.3% (0.5%)	45.0% (0.6%)	48.3%	48.5%	49.2%
Employed	55.1% (0.4%)	54.7% (0.5%)	55.0% (0.6%)	51.7%	51.5%	50.8%
**Longstanding illness**						
No	57.4% (0.4%)	54.3% (0.5%)	53.0% (0.6%)	53.7%	50.7%	48.6%
Yes	42.6% (0.4%)	45.7% (0.5%)	47.0% (0.6%)	46.3%	49.3%	51.4%
**Current smoking**						
Non-smoker	75.2% (0.3%)	79.2% (0.4%)	84.1% (0.5%)	75.9%	79.8%	85.6%
Current smoker	24.8% (0.3%)	20.8% (0.4%)	15.9% (0.5%)	24.1%	20.2%	14.4%
**Current drinking**						
No	19.6% (0.3%)	22.2% (0.4%)	22.6% (0.6%)	21.1%	23.6%	23.3%
Yes	80.4% (0.3%)	77.8% (0.4%)	77.4% (0.6%)	78.9%	76.4%	76.7%
**Equivalized household income**						
Top quintile	22.9% (0.5%)	21.9% (0.6%)	22.1% (0.7%)	21.3%	21.0%	21.8%
2nd	21.6% (0.4%)	21.5% (0.5%)	21.7% (0.7%)	21.1%	20.9%	21.3%
3rd	19.7% (0.4%)	20.3% (0.5%)	18.8% (0.6%)	20.0%	20.3%	19.5%
4th	18.9% (0.4%)	17.9% (0.5%)	19.1% (0.7%)	20.2%	19.3%	19.5%
Bottom quintile	16.9% (0.4%)	18.4% (0.5%)	18.4% (0.6%)	17.4%	18.5%	17.9%
**Area deprivation**						
1st—most deprived	19.4% (0.6%)	19.0% (0.7%)	18.4% (0.9%)	18.9%	16.8%	15.5%
2nd	19.9% (0.6%)	20.4% (0.6%)	20.6% (0.8%)	19.0%	19.7%	19.5%
3rd	19.8% (0.5%)	19.8% (0.6%)	19.4% (0.7%)	21.5%	22.4%	20.7%
4th	21.1% (0.6%)	20.2% (0.6%)	20.6% (0.7%)	22.6%	22.0%	22.7%
5th—least deprived	19.9% (0.7%)	20.6% (0.7%)	21.1% (0.9%)	18.1%	19.1%	21.7%
**Weekly drinking category**						
Non-drinker	13.7% (0.3%)	16.2% (0.3%)	16.4% (0.5%)	14.7%	17.2%	16.9%
Moderate	63.8% (0.4%)	64.6% (0.4%)	65.2% (0.6%)	64.3%	64.2%	65.2%
Hazardous	18.0% (0.3%)	15.4% (0.3%)	15.0% (0.4%)	16.9%	15.0%	14.8%
Harmful	4.5% (0.2%)	3.8% (0.2%)	3.4% (0.2%)	4.1%	3.6%	3.1%
**Weekly consumption of alcohol (UK standard units)**						
Mean (SD)	11.9 (0.2)	10.4 (0.2)	9.8 (0.2)	11.04 (19.01)	9.97 (17.22)	9.44 (16.17)

### 3.2. Multivariate regressions for drinking in Scotland

#### 3.2.1. Drinking categories

Table 2 shows changes in the likelihood of drinking categories in Scotland that occurred after 2012 and post MUP’s introduction in 2018. The overall drinking prevalence declined significantly and consecutively after the announcement in 2012 (OR = 0.85, p<0.001) and enactment in 2018 (OR = 0.84, p<0.0001). The likelihood of heavy drinking (at hazardous or harmful level) also reduced significantly after 2012 with adjusted OR = 0.88 (p<0.001) and continued to drop in 2018 with OR = 0.87 (p<0.001). Similar observations were seen for harmful drinking where in comparison to the period 2008–2012, the probability of harmful drinking is about 0.95 during 2012–2017 and dropped further to 0.98 after 2018, although these reductions were not significant (p>0.10). Even after excluding observations post COVID-19, the results showed consistently that a statistically change was observed after 2012 whilst the change pre- and post-2018 was much less (see S4 File). These findings in summary suggested a reduction of drinking across all drinking categories starting in 2012.

**Table 2:** Generalized ordered logistic models for drinking categories in Scotland, 2008–2021.

Drinking category	Current drinking ^a^	Heavy drinking ^b^	Harmful drinking ^c^
**Period**			
*2008–2012*	Ref	Ref	ref
*2013–2017*	0.85 (0.03) ***	0.88 (0.03) ***	0.95 (0.06)
*2018–2021*	0.84 (0.04) ***	0.87 (0.03) ***	0.89 (0.08)
**Male**	1.30 (0.04) ***	1.42 (0.03) ***	1.54 (0.08) ***
**Age**	0.99 (0.00) ***	1.00 (0.00)	1.00 (0.00) *
**Ethnic (White)**	7.74 (0.65) ***	3.97 (0.49) ***	4.94 (1.31) ***
**Married/ cohabited**	1.23 (0.04) ***	0.98 (0.03)	0.84 (0.05) **
**High education**	1.41 (0.05) ***	1.15 (0.03) ***	0.91 (0.06)
**Employed**	1.73 (0.07) ***	1.08 (0.04) **	0.86 (0.05) **
**Current smoking**	1.18 (0.05) ***	1.94 (0.06) ***	2.62 (0.16) ***
**Long term illness**	0.67 (0.02) ***	0.90 (0.03) **	1.14 (0.07) **
**Equivalized income**			
*Top quintile*	Ref	Ref	ref
*2nd*	0.84 (0.06) **	0.81 (0.03) ***	0.89 (0.08)
*3rd*	0.62 (0.04) ***	0.70 (0.03) ***	0.78 (0.08) **
*4th*	0.54 (0.03) ***	0.55 (0.03) ***	0.62 (0.06) ***
*Bottom quintile*	0.46 (0.03) ***	0.52 (0.03) ***	0.82 (0.08) **
**Area deprivation**			
*1st—Most deprived*	Ref	Ref	ref
*2nd*	1.14 (0.06) **	1.03 (0.05)	0.93 (0.08)
*3rd*	1.42 (0.08) ***	1.15 (0.06) **	1.06 (0.10)
*4th*	1.38 (0.08) ***	1.11 (0.06) **	1.00 (0.10)
*5th–Least deprived*	1.50 (0.10) ***	1.30 (0.07) ***	0.95 (0.09)

N = 53,719.

*** p<0.001

** p<0.05

*p<0.10

Odds ratios (SE) were reported after weighting for complex survey design.

a—Current drinking: abstainer (ref) vs. moderate/ hazardous/ harmful drinking.

b—Heavy drinking: abstainer / moderate (ref) vs. hazardous/ harmful drinking.

c—Harmful drinking: abstainer / moderate/ hazardous (ref) vs. harmful drinking.

For the overall drinking, those who were married or currently cohabiting had a higher probability of current drinking (OR = 1.23, p<0.001) but were less likely to engage in harmful drinking (OR = 0.84, p<0.05) or heavy drinking (OR = 0.98, p>0.10). People with higher education level or employed showed a higher chance of current drinking as well as heavy drinking but were less likely to exhibit harmful drinking (OR for harmful drinking among high educated was 0.91, and among employed was 0.86, p<0.05 in both). Males and current smokers were more likely to drink in general, but they also had a much higher probability of drinking at heavy or harmful levels.

#### 3.2.2. Total alcohol consumption

Table 3 shows the estimated decline in quantity consumed after the announcement in 2012 and the enactment in 2018 of MUP from negative binomial model. As can be seen, the drinking amount dropped overall but mostly among those drinking at moderate levels. After two consecutive policy changes from late 2011 to mid-2012, the average weekly drinking amount reduced about 8% (p = 0.068) and the consumption during 2018–2021 reduced even further at 12% (p = 0.019) as compared to the average consumption in 2008–2011. The decline was equivalent to the estimated margins of alcohol consumption dropping from 10.51 units per capita per week (2008–2012) to 9.70 in (2013–2017) to further 9.35 units in 2018–2021. Although the decrease was significant in the overall population, the reduction in the amount consumed was only significant among moderate drinkers after the enactment in 2018. After the policy changes happened, the period from 2013–2017 did see a decline in consumption in all three drinking categories but these changes were not significant (even at p = 0.10). Similar findings were also shown in the additional analysis after excluding the observations after 2020 (see S4 File).

**Table 3:** Negative binomial models on the weekly drinking amount in Scotland, 2008–2021.

Alcohol consumption	Overall ^a^	Moderate drinkers ^b^	Hazardous drinkers ^c^	Harmful drinkers ^d^
**Period**				
*2008–2012*	**Ref**	**Ref**	**ref**	**ref**
*2013–2017*	**-0.08 (0.04)** *	**-0.03 (0.03)**	**-0.02 (0.02)**	**-0.02 (0.07)**
*2018–2021*	**-0.12 (0.05)** **	**-0.11 (0.03)** **	**-0.01 (0.02)**	**0.02 (0.07)**
**Male**	0.66 (0.03)***	0.56 (0.02)***	0.39 (0.01)***	0.37 (0.06)***
**Age**	0.00 (0.00)*	0.00 (0.00)**	0.00 (0.00)	-0.01 (0.00)**
**Ethnic (White)**	0.86 (0.18)***	0.44 (0.11)***	-0.05 (0.07)	0.00 (0.13)
**Married/ cohabited**	-0.03 (0.04)	0.05 (0.03)*	-0.01 (0.02)	-0.24 (0.06)***
**High education**	0.10 (0.04)**	0.07 (0.03)**	-0.02 (0.02)	-0.10 (0.07)
**Employed**	0.04 (0.05)	0.10 (0.03)**	0.01 (0.02)	-0.08 (0.05)
**Current smoking**	0.42 (0.05)***	0.13 (0.04)***	0.03 (0.02)*	0.05 (0.07)
**Long term illness**	-0.04 (0.04)	-0.12 (0.03)***	0.01 (0.01)	0.03 (0.05)
**Equivalized income**				
*Top quintile*	Ref	ref	ref	ref
*2nd*	-0.11 (0.05)**	-0.11 (0.04)**	0.00 (0.02)	0.05 (0.10)
*3rd*	-0.19 (0.05)***	-0.14 (0.04)***	-0.01 (0.02)	-0.10 (0.10)
*4th*	-0.34 (0.06)***	-0.22 (0.04)***	-0.08 (0.03)**	-0.04 (0.12)
*Bottom quintile*	-0.30 (0.08)***	-0.26 (0.05)***	0.03 (0.03)	0.01 (0.16)
**Area deprivation**				
*1st—Most deprived*	Ref	ref	ref	ref
*2nd*	-0.02 (0.07)	-0.01 (0.05)	0.02 (0.02)	-0.02 (0.08)
*3rd*	0.12 (0.07)	0.03 (0.05)	0.02 (0.02)	0.18 (0.13)
*4th*	0.03 (0.07)	0.09 (0.05)*	0.01 (0.02)	0.07 (0.09)
*5th–Least deprived*	0.14 (0.07)*	0.13 (0.05)**	0.01 (0.03)	0.02 (0.09)
**Margins of drinking amount (UK standard units)**				
**Period**				
**2008–2012**	**10.51 (0.34)**	**5.51 (0.12)**	**25.92 (0.29)**	**72.19 (4.05)**
**2013–2017**	**9.70 (0.29)**	**5.34 (0.12)**	**25.50 (0.29)**	**70.51 (2.49)**
**2018–2021**	**9.35 (0.36)**	**4.96 (0.13)**	**25.56 (0.40)**	**73.66 (4.57)**

a Overall N = 53,347

b Moderate drinkers = 34,721

c Hazardous drinkers = 8,669

d Harmful drinkers = 1,988

.*** p<0.001

** p<0.05

* p<0.10. Coefficients (SE) were weighted for complex survey designs. Margins in the three bottom rows were estimated from negative binomial models.

### 3.3. Difference-in-difference models

Table 4 shows the 2x2 DID (unmatched) models for the change in drinking rate in Scotland relative to the two other control groups. All four models show a reduction in current drinking with the negative coefficients of DID interaction term. However, model 1 with Northern Ireland is the only model showing a significant DID impact after p 2012 (p = 0.001) while the comparison with England during the same time frame was not significant (p = 0.574). The other two DID models also comparing with England estimated the change before and after MUP’s enactment were also not significant with p>0.05.

**Table 4:** The 2x2 DID models of current drinking rate.

	Coef.	SE	P value	95% CI	
**Model 1: Northern Ireland vs Scotland (2011–2012 vs 2013–2015)**					
After MUP’s announcement	0.018	0.008	0.020	0.003	0.033
Scotland	0.081	0.007	0.000	0.066	0.094
DID interaction term	-0.032	0.009	0.001	-0.050	-0.014
**Model 2: England vs Scotland (2011–2012 vs 2013–2015)**					
After MUP’s announcement	-0.013	0.006	0.043	-0.025	0.000
Scotland	-0.009	0.005	0.097	-0.019	0.002
DID interaction term	-0.005	0.008	0.574	-0.021	0.012
**Model 3: England vs Scotland (2016–2017 vs 2018–2019)**					
After MUP’s enactment	-0.022	0.008	0.006	-0.037	-0.006
Scotland	0.002	0.007	0.761	-0.011	0.015
DID interaction term	-0.006	0.011	0.615	-0.027	0.016
**Model 4: England vs Scotland (2013–2017 vs 2018–2019)**					
After MUP’s enactment	-0.019	0.004	0.000	-0.028	-0.011
Scotland	0.001	0.005	0.785	-0.009	0.012
DID interaction term	-0.007	0.009	0.410	-0.025	0.010

Using the Kernel matching, Table 5 provides the estimated effect of MUP on current drinking in Scotland after 2012 and the enactment in 2018. For Northern Ireland, the current drinking rate increased from 2011–2015 while in Scotland it reduced, hence, after matching, the actual reduction in drinking rate attributed to MUP was estimated at 5.8% (SE = 0.9%, p<0.001) (see model 1). In model 2 between England and Scotland, as both countries observed a decline in drinking rate from 2011–2015, the reduction in drinking rate attributed to MUP was estimated at 2.3% (SE = 0.7%, p = 0.001). With adjustment for survey weight, the estimation of MUP impact was calculated at about 2.6% (SE = 0.7%, p<0.001). Taken together, the two DID models with two different control groups suggest that the joint impact of policy changes by end of 2012 had significantly reduced current drinking rate in Scotland compared with NI and England.

**Table 5:** Kernel PSM-DID models for the introduction of MUP in 2012.

**MODEL 1**	Naïve estimates							
	**Outcome**	**SE**	**t-stat**	**P**				
**2011–2012**								
Northern Ireland	69.8%							
Scotland	79.7%							
*Difference*	10.0%	0.70%	14.82	<0.001				
**2013–2015**								
Northern Ireland	72.3%							
Scotland	76.5%							
*Difference*	4.1%	0.60%	6.95	<0.001				
**Diff-in-Diff**	**-5.8%**	**0.90%**	**6.49**	**<0.001**				
**MODEL 2**	**Naïve estimates**				**Survey weighted**			
	**Outcome**	**SE**	**t-stat**	**P**	**Outcome**	**SE**	**t-stat**	**P**
**2011–2012**								
England	82.1%				82.2%			
Scotland	79.6%				79.7%			
Difference	-2.4%	0.5%	-4.82	<0.001	-2.4%	0.5%	-4.84	<0.001
**2013–2015**								
England	81.2%				81.4%			
Scotland	76.5%				76.5%			
Difference	-4.8%	0.4%	10.67	<0.001	-5.0%	0.4%	11.2	<0.001
**Diff-in-Diff**	**-2.3%**	**0.7%**	**3.46**	**0.001**	**-2.6%**	**0.7%**	**3.8**	**<0.001**

In Table 6, model 3 shows that the estimation of MUP impact after the enactment in 2018 following the examined period in model 2. With England as the control group, the naïve estimation of DID impact was about 2.8% (SE = 0.8%, p = 0.001) and about 3.2% (SE = 0.8%, p<0.001) if adjustment is made for survey weights. Extending the baseline period to 2013–2017 (from announcement to enactment), model 4 shows similar results of DID estimations with the naïve estimation of 2.5% (SE = 0.5%, p<0.001) and the weighted estimation of 2.6% (SE = 0.6%, p<0.001). Both model 3 and model 4 indicate a significant reduction in current drinking rate associated with MUP before and after enactment in 2018 in Scotland.

**Table 6:** Kernel PSM-DID model for the enactment of MUP in 2018.

**MODEL 3**	**Naïve estimates**				**Survey weighted**			
	**Outcome**	**SE**	**t-stat**	**p**	**Outcome**	**SE**	**t-stat**	**P**
**2016–2017**								
England	81.1%				81.0%			
Scotland	78.4%				78.6%			
*Difference*	-2.6%	0.6%	-4.28	<0.001	-2.4%	0.6%	-3.89	<0.001
**2018–2019**								
England	81.4%				81.5%			
Scotland	75.9%				75.9%			
*Difference*	-5.4%	0.5%	10.37	<0.001	-5.5%	0.5%	10.57	<0.001
**Diff-in-Diff**	**-2.8%**	**0.8%**	**3.47**	**0.001**	**-3.2%**	**0.8%**	**3.9**	**<0.001**
**MODEL 4**	**Naïve estimates**				**Survey weighted**			
	**Outcome**	**SE**	**t-stat**	**p**	**Outcome**	**SE**	**t-stat**	**P**
**2013–2017**								
England	81.2%				81.3%			
Scotland	78.2%				78.3%			
*Difference*	-3.0%	0.4%	-8.2	<0.001	-3.0%	0.4%	-8.23	<0.001
**2018–2019**								
England	81.4%				81.5%			
Scotland	75.9%				75.9%			
*Difference*	-5.4%	0.5%	10.33	<0.001	-5.5%	0.5%	10.54	<0.001
**Diff-in-Diff**	**-2.5%**	**0.6%**	**3.87**	**<0.001**	**-2.6%**	**0.6%**	**4.030**	**<0.001**

## 4. Discussion

In this study, the impact of the MUP policy was examined in two interdependent analytical steps: the first examined activity in Scotland only, the second examined activities in Scotland in a comparative analysis with Northern Ireland and England as a way of adjusting for secular trends. Each analysis possesses technical strengths and drawbacks but, collectively, they provide compelling evidence that MUP has impacted on alcohol consumption in Scotland in a manner that may improve public health.

### 4.1. Drinking behaviour in Scotland upon the introduction of MUP

All available indicators of drinking behaviour point to positive changes in the pattern of drinking since 2012 and these changes continued after 2018 when the MUP policy was enacted. These findings are in keeping with the results of recent studies about the impact of MUP in Scotland [6,7,11,13–18,33].

Firstly, regarding drinking versus abstinence, a reduction in the overall drinking rate in Scotland was observed with similar patterns were observed for heavy drinkers which combined those who drank at either hazardous or harmful levels. Even though it should be noted that the prevalence of harmful drinking did decrease but the margin of change was not statistically significant. Existing literature provided evidence regarding alcohol consumption levels that echo this study’s findings [6,15,18,33]. Stevely A.K. et al (2023) using Kantar Alcovision survey to compare Scotland with Northern England found no changes in prevalence of harmful drinking or moderate drinking but a significant reduction of 3.5% in the hazardous drinking group [33], which is similar to our findings. A recent review in 2023 suggested that consumption reduced among those exceeding the UK low risk drinking guidelines as the current drinking rate was found to reduce in this study [6]. Together, this suggests an impact of MUP on drinking overall and small changes among heavy drinkers whom the Scottish Government sought to target with this policy. The MUP policy had already shown some positive changes among the harmful drinkers which while not significant are encouraging considering their fairly small population share (less than 5% in this study) and higher risks of alcohol dependence [34,35]. As more data emerges, further analysis on this subgroup seems warranted.

Secondly, the investigation into the amount of alcohol consumed lends another perspective to the impact of MUP on drinking behaviour in Scotland. The weighted statistics of weekly alcohol consumption in Scotland showed a significant reduction in the amount consumed since the introduction of MUP and subsequently with the policy enactment six years later. Immediately after 2012, the total weekly consumption has been estimated to fall about 8% compared to the average level before MUP announcement. After 2018, the total consumption was estimated to drop even further by 11% compared to the average level of 2008–2011. This is equivalent to an estimated reduction of 0.81 unit of alcohol after MUP’s introduction with an additional decline of 0.36 units after MUP’s enactment. This result is consistent with previous studies that investigated alcohol consumption using market data sources [11,13–15, 18]. A number of studies using aggregated alcohol sales data showed a reduction in alcohol sales by 3.0% to 3.5% with major changes seen in cider and spirits [6,11,13,14]. Other studies using Kantar household shopping panel data also showed that weekly alcohol purchases per adult in Scotland reduced around 6.2% [18] to 7.1% [17] compared to England between the period 2015-mid 2018 and the latter half of 2018 after the introduction of MUP.

From another perspective, the changes in overall drinking and the amount consumed after 2012 was more prominent than the decline observed after 2018. This finding was further supported by our additional investigation on the time-trend of drinking behavior (S3 File) which showed that the decline of current drinking rates after 2012 was statistically significant while the decrease trend appeared from 2013 onward was not. This could be explained as pre-2012 Scottish government has taken two policy actions consecutively both aimed to suppress alcohol purchasing: a multi-buy discount ban (October 2011) and government’ intention to implement MUP (June 2012). Perhaps, if it was not due to the repeated legal challenges led by the Scotch Whisky Association from 2012 to 2017, the impact of MUP’s announcement would have been superseded by that of its enactment though this is speculation. Disentangling the impact of the announcement from the almost contemporaneous ban on quantity discounts with annual survey data is problematic. Attribution of effect within a real world context where policies evolve, effects accumulate, potentially with a lag makes attribution challenging underscoring the need for a more thoughtful and rigorous analytic approach than might otherwise be the case.

Otherwise, the statistics on amount of alcohol consumption were found to decline for the overall population but the actual change in drinking amount was found significant only among moderate drinkers and not for hazardous and harmful drinkers. For hazardous and harmful drinker, even though the decline was not statistically significant, the pattern of decline immediately after 2012 showed recovery back to the pre-MUP period suggesting a shock influence of MUP when it was first introduced especially for heavy drinkers. This finding could be interpreted as the policy impact of MUP in conjunction with other policies might not yet achieve a sustained desired impact but did serve to act as a reminder of the harms of drinking to those perhaps most receptive to such messages–moderate drinkers.

In summary, the examination of changes in Scotland suggests that the introduction and implementation of MUP subsequently influenced drinking behaviours both in terms of whether to drink or not and in terms of the level consumed (either in quantity or by hazardous level). Within the context of an overall policy environment increasingly hostile to alcohol, there may be uncertainty on whether harmful drinking was curtailed, where the relatively small sample and the relatively short follow-up after the MUP’s enactment are limiting factors in this study and where further research is warranted.

### 4.2. Difference-in-difference analysis of policy impact of MUP in Scotland

Further comparative analysis confirmed the impact of MUP on alcohol behaviours by comparing the change pre- and post- MUP situation between Scotland and England and Northern Ireland in both matched and unmatched DID analysis. The findings from DID consistently confirm that overall drinking fell after MUP, as seen from the results from uncontrolled design above.

Firstly, for the introduction of MUP, Scotland showed a reduction of about 5.8% for the matched sample relative to drinking rate in Northern Ireland and about 2.3% with England as control group. The DID estimations differed for two control groups for two main reasons. The first reason is due to the entirely opposite change of drinking in Scotland (decreasing over time) and Northern Ireland (increased overall from 2011). While a MUP policy was mooted in England as in Scotland in 2012, England dropped the plan shortly afterwards. However, the brief announcement in England may have had an effect that served to dampen the difference observed with Scotland. Secondly, the rate of current drinking in England and Scotland were quite similar which suggested that unless a marked impact was imposed, the changes in alcohol behaviours might be missed (see S1 Fig in S2 File).

Moving onto the enactment of MUP in 2018, two DID analyses of Scotland versus England further emphasized the significant impact of MUP adoption on the drinking rate. After matching the Scotland’ sample with England, the average difference in drinking rate before and after MUP implementation was estimated at 3.2% between 2016–2017 vs. 2018–2019. If the baseline period was extended from 2013–2017, the average difference due to MUP was slightly smaller at 2.6% which may be due to the additional influence from other alcohol policies adopted in England and Scotland during this time window. In 2014, Scotland had adopted a reduction in the drink-drive limit [5] and in England bans on selling heavily discounted alcohol by imposing a “permitted price” (defined by alcohol duty plus VAT) to all licensed premises [36]. But above all, the DID analysis continued to show a significant reduction in overall drinking rate in Scotland under the enactment of MUP in Scotland.

Aside from the results obtained in the matched DID, the unmatched DID analysis also showed the reduction of current drinking rate upon the introduction of MUP either during the announcement or the enactment, although the reduction was fairly small and insignificant (except for Northern Ireland). This finding should not be overlooked as the MUP’s impact was theoretically limited to the “entry” and “exit” category of drinking, leaving a reservoir of “social drinkers” on whom MUP had little or no effect in the sense that drinking per se remained only slightly changed. In addition to that, one should consider differences in alcohol related policies adopted in Scotland, England and Northern Ireland which seems likely to violate the parallel trend assumption for DID analysis. Hence, the results of unmatched DID must be interpreted with caution given this limitation and that the findings from the PSM-DID has limited in generalizability.

### 4.3. Study limitations

The study findings should be interpreted with a degree of caution. The biggest drawback of this study lies within the data sources employed to evaluate the policy impacts. The first component examined changes solely in Scotland hence the causal inference for the findings is weaker when using uncontrolled design. The second component–although employing a more rigorous design for evaluating policy effectiveness–was limited by the scope of control or comparator groups. The available data for DID analysis limited the comparison to within one year after MUP implementation and for one outcome–current drinking. Current drinking is a blunt indicator for alcohol drinking behaviour, given that it does not differentiate between problem and non-problem drinking nor examine the amounts consumed [37]. In addition to that, MUP policy is designed to preserve price increases in the cheapest priced alcohol, target heavy and frequent consumers of cheap alcohol. Curbing social drinking might be a low priority for MUP. From a broader perspective, one should also consider the incremental and multi-pronged approach of Scottish Government policy to alcohol, which was likely to provide a cumulative effect during the 6-year lead time to enactment of MUP from its announcement from 2012–2018 and complicate the decomposition of MUP from policies adopted before and afterwards. This might produce a degree of confounding in societal attitudes towards alcohol consumption on the estimation of the true policy impact. Alcohol consumption and heavy drinking in UK reached a peak in mid-2000s and fell steadily since then with public health campaigns on the health effects of alcohol becoming more widespread during the sample period [38,39]. Given these drawbacks, further studies about drinking behaviour, making use of data that allow greater discrimination in time than an annual survey are highly encouraged to validate the results presented in this study.

Another limitation is due to the weakness in methodology regarding the three surveys used in this study. First, these datasets are not panel data and did not allow tracking of individuals’ drinking behaviour over time. Furthermore, population-based household survey data are likely to under-represent both general consumption levels and specifically heavier drinkers due to the lack of participation in self-report surveys. In this analysis, the sample of heavy drinkers was small (perhaps reflecting selection effects and under-reporting) which may have impacted on statistical power. Otherwise, the study complements previous studies that used a controlled interrupted time series design on hospital data or market purchasing data [6,7,11,13–18,33], by providing results based on a representative sample and controlled for socio-demographic factors.

It should also be noted that while all three surveys were designed to be broadly representative and allow examination of the same issue, they are not identical and caution must be exercised in respect of the conclusions that may be drawn given methodological differences in how these surveys are conducted. SHeS and HSE have similarities in terms of the survey flow and questionnaire structure, but differences might exist with respect to sampling method, survey procedure and organization of data collection. In addition, selection and reporting bias provide opportunities for differences to exist that are not solely attributable to the policy studied. The same caution was noted for Northern Ireland data given the entirely different survey panels to the Scotland or England survey panel.

Given structural inequalities and their relationships with risky behaviour and health inequalities, future research should examine distributional effects of MUP, an analysis only possible using survey data given the need to characterize the socio-demographic profile of individuals. Further investigation might offer insights into how MUP might affect more change among poor people or among women, and better validation might be achieved if a similar dataset is available for control or comparator groups.

## 5. Conclusion

This study offers evidence that the announcement and enactment of MUP in Scotland produced changes in drinking behaviours consistent with efforts to improve public health. The analyses suggested that there was a reduction in the amount of alcohol consumed, and in the prevalence of overall drinking as well as heavy drinking, but as yet, not to a significant rate or significant decrease in drinking amount by harmful drinkers. Although the changes that occurred after MUP adoption may not have been as strong as expected, a positive shift in social drinking in Scotland was captured which may be deemed laudable in public health terms. The presence of a MUP policy–and ongoing discussion about MUP–may serve to encourage a wider debate about its extension with respect to alcohol reduction in degree or in terms of other jurisdictions. The use of this fiscal instrument may also be worth considering in respect to consumption of other food and drink items which may harm public health.

## Supporting information

S1 File Description of included variables. (PDF)

S2 File Descriptive analysis of Scotland versus Northern Ireland and England. (PDF)

S3 File Preliminary investigation of the time trend of drinking rate in Scotland. (PDF)

S4 File Additional examination of drinking pattern in Scotland from 2008–2019 (excluding data post-COVID). (PDF)
