# Supplementary material for: An investigation into patterns of Alcohol drinking in Scotland after the introduction of minimum unit pricing
Source: PLoS One. 2024 Aug 1;19(8):e0308218. doi: 10.1371/journal.pone.0308218 (PMC11293661; doi:10.1371/journal.pone.0308218)
Supplement: S1 File — (PDF) [file pone.0308218.s001.pdf]

Supplementary 1: Description of included variables

Table S1: Selected variables of each dataset in the final analysis

Variables	Scotland	N.Ireland	England	Description
Sex	✓	✓	✓	1= Male; 2 = Female
Age	✓	✓	✓	Scotland & England: Continuous variable from 16-90+.<br>Northern Ireland: grouped as 16-19; 20-24; 25-34; 35-49; 50-59; 60+.
Marital status	✓	✓	✓	1 = Married/ cohabited<br>0 = Single/ Separated/ Divorced/ Widowed
Ethnic	✓		✓	1= White<br>0 = Others/ Mixed
Education	✓	✓	✓	1 = High education (*Scotland: HNC/D and above; Northern Ireland: Vocational training/ bachelor's degree and above; England: Higher education above NQV3/ GCE-A level*)<br>0 = Lower education.
Employment	✓	✓	✓	1 = Employed<br>0 = ILO unemployed/ Retired/ Economically inactive
Self-rated health	✓	✓	✓	1 = Very good/ Good; 2 = Fair; 3 = Bad/ very bad
Depression/ anxiety	✓			1 = having any depression or anxiety symptoms<br>0 = no symptoms of depression or anxiety
Long-term illness	✓	✓	✓	1 = Yes<br>0 = No
Smoking	✓	✓	✓	1 = Current smoker; 0 = Former/ never-smoker
Current drinking	✓	✓	✓	1 = Current drinking<br>0 = Never/ Very occasionally
Drinker category	✓			1 = Non-drinker (never drinking/ drinking very occasional)<br>2 = Moderate (men: ≤21 units; women: ≤14 units)<br>3 = Hazardous (men: over 21-50; women: over 14-35)<br>4 = Harmful (men over 50/ women over 35)
Drinking amount	✓			Total alcohol consumption per week in UK standard units
Income	✓	✓	✓	Household income quintiles from 1 (richest) to 5 (poorest).<br>In Northern Ireland**,** data was not equivalized
Deprivation	✓		✓	5 quintiles from 1st (most deprived) to 5th (least deprived)
