# Supplementary material for: An investigation into patterns of Alcohol drinking in Scotland after the introduction of minimum unit pricing
Source: PLoS One. 2024 Aug 1;19(8):e0308218. doi: 10.1371/journal.pone.0308218 (PMC11293661; doi:10.1371/journal.pone.0308218)
Supplement: S2 File — (PDF) [file pone.0308218.s002.pdf]

Supplementary 2: Descriptive analysis of Scotland versus Northern Ireland and England

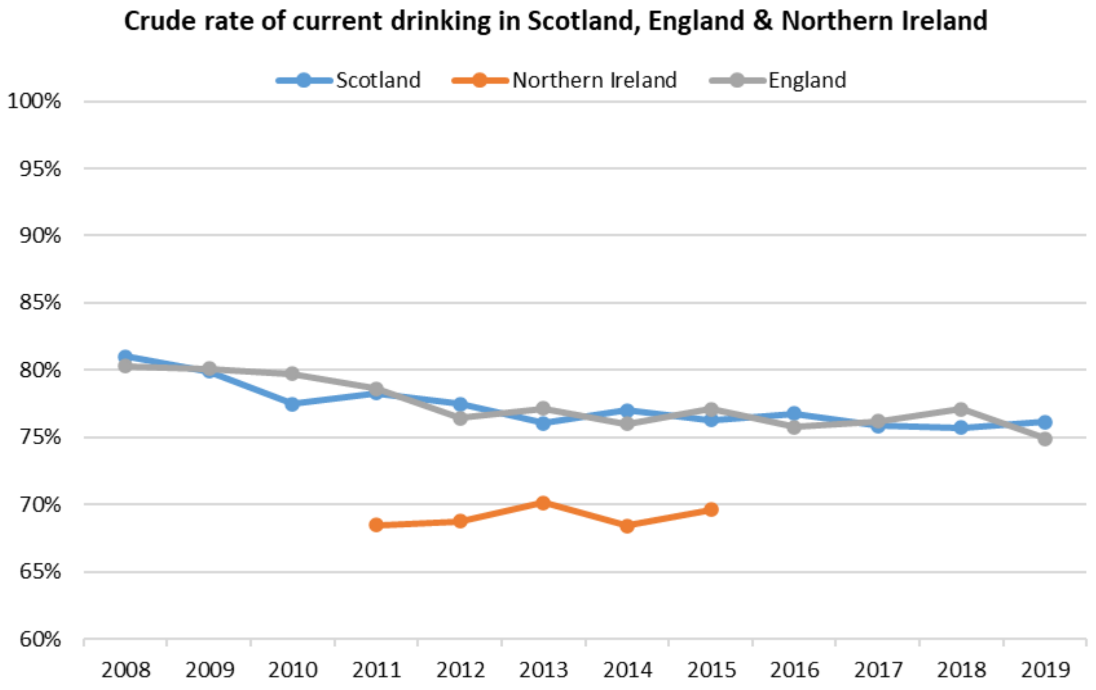

Figure S1: Crude rate of current drinking in Scotland, England & Northern Ireland

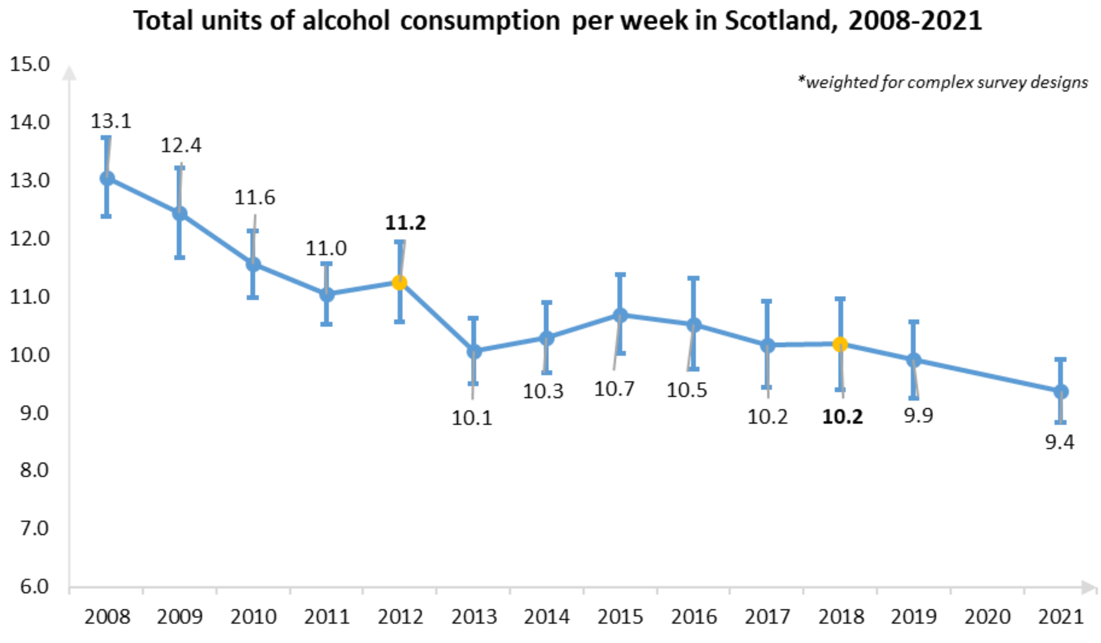

Figure S2: The average alcohol consumption per week in Scotland, 2008-2021 (weighted for complex survey design)

Table S1: Descriptive analysis of Scotland vs. Northern Ireland, 2011-2015 (DID model 1)

	2011-2012			2013-2015		
	**N. Ireland**	**Scotland**	**p**	**N. Ireland**	**Scotland**	**p**
**N**	9,685	12,359		10,733	14,550	
**Sex**			<0.001			0.026
Female	53.1%	56.2%		57.0%	55.6%	
Male	46.9%	43.8%		43.0%	44.4%	
**Age groups**			<0.001			<0.001
16-19	5.6%	3.6%		3.5%	3.6%	
20-24	7.4%	5.0%		5.2%	5.3%	
25-34	17.4%	12.3%		15.3%	13.1%	
35-49	26.9%	26.0%		26.8%	23.9%	
50-59	16.3%	17.8%		17.1%	18.1%	
60+	26.4%	35.3%		32.2%	36.1%	
**Marital status**			<0.001			<0.001
Single/ Divorced/ Separated/ Widowed	42.9%	36.7%		46.4%	38.1%	
Married/ co-habited	57.1%	63.3%		53.6%	61.9%	
**Education**			<0.001			<0.001
Lower educated	52.1%	62.2%		63.2%	59.6%	
Higher educated	47.9%	37.8%		36.8%	40.4%	
**Employment**			0.35			0.51
Unemployed	49.5%	48.9%		48.8%	48.3%	
Employed	50.5%	51.1%		51.2%	51.7%	
**Longstanding illness**			<0.001			<0.001
No	66.8%	51.5%		63.4%	51.1%	
Yes	33.2%	48.5%		36.6%	48.9%	
**Current smoking**			0.12			0.35
Non-smoker	76.0%	77.0%		78.6%	79.0%	
Current smoker	24.0%	23.0%		21.4%	21.0%	
**Current drinking**			<0.001			<0.001
No	31.4%	22.0%		30.6%	23.5%	
Yes	68.6%	78.0%		69.4%	76.5%	
**Household income**			<0.001			<0.001
Top quintile	20.4%	22.5%		18.5%	21.0%	
2nd	20.0%	21.4%		15.3%	21.0%	
3rd	22.6%	19.6%		19.7%	20.4%	
4th	16.9%	19.3%		13.0%	19.2%	
Bottom quintile	20.1%	17.3%		33.5%	18.3%	

Table S2: Descriptive analysis of Scotland versus England, 2011-2015 (DID model 2)

	2011-2012			2013-2015		
	**England**	**Scotland**	**p**	**England**	**Scotland**	**p**
**N**	16,900	12,359		24,906	14,550	
**Sex**			0.28			0.76
Female	55.6%	56.2%		55.5%	55.6%	
Male	44.4%	43.8%		44.5%	44.4%	
**Age groups**			<0.001			<0.001
16-19	4.4%	3.6%		4.5%	3.6%	
20-24	5.6%	5.0%		5.0%	5.3%	
25-34	14.5%	12.3%		14.3%	13.1%	
35-49	26.0%	26.0%		26.1%	23.9%	
50-59	15.9%	17.8%		16.2%	18.1%	
60+	33.5%	35.3%		34.0%	36.1%	
**Marital status**			0.71			<0.001
Single/ Divorced/ Separated/ Widowed	36.5%	36.7%		35.9%	38.1%	
Married/ co-habited	63.5%	63.3%		64.1%	61.9%	
**Education**			<0.001			<0.001
Lower educated	64.9%	62.2%		63.7%	59.6%	
Higher educated	35.1%	37.8%		36.3%	40.4%	
**Employment**			<0.001			<0.001
Unemployed	46.9%	48.9%		46.2%	48.3%	
Employed	53.1%	51.1%		53.8%	51.7%	
**Longstanding illness**			<0.001			<0.001
No	57.4%	51.5%		57.6%	51.1%	
Yes	42.6%	48.5%		42.4%	48.9%	
**Current smoking**			<0.001			<0.001
**Non-smoker**	80.5%	77.0%		81.8%	79.0%	
**Current smoker**	19.5%	23.0%		18.2%	21.0%	
**Current drinking**			0.40			0.48
No	22.4%	22.0%		23.2%	23.5%	
Yes	77.6%	78.0%		76.8%	76.5%	
**Household income**			<0.001			0.014
Top quintile	20.3%	22.5%		22.2%	21.0%	
2nd	21.6%	21.4%		21.4%	21.0%	
3rd	19.9%	19.6%		20.4%	20.4%	
4th	19.7%	19.3%		18.0%	19.2%	
Bottom quintile	18.5%	17.3%		18.1%	18.3%	
**Area deprivation**			<0.001			<0.001
1st - most deprived	18.0%	17.8%		19.0%	17.1%	
2nd	18.9%	17.9%		18.9%	20.4%	
3rd	20.7%	23.1%		20.4%	22.4%	
4th	21.2%	22.6%		20.5%	22.0%	
5th - least deprived	21.3%	18.5%		21.1%	18.2%	

Table S3: Descriptive analysis of Scotland versus England, 2016-2019 (DID model 3)

	2016-2017			2018-2019		
	**England**	**Scotland**	**p**	**England**	**Scotland**	**p**
**N**	16,008	8,020		16,382	9,713	
**Sex**			0.28			0.051
Female	55.7%	56.5%		55.2%	56.4%	
Male	44.3%	43.5%		44.8%	43.6%	
**Age groups**			<0.001			<0.001
16-19	3.8%	3.1%		4.2%	3.4%	
20-24	4.8%	5.1%		4.8%	4.2%	
25-34	14.3%	13.0%		13.7%	12.2%	
35-49	24.4%	22.3%		24.7%	22.4%	
50-59	17.5%	19.1%		17.4%	19.0%	
60+	35.2%	37.3%		35.2%	38.8%	
**Marital status**			0.015			<0.001
Single/ Divorced/ Separated/ Widowed	35.1%	36.7%		35.2%	37.5%	
Married/ co-habited	64.9%	63.3%		64.8%	62.5%	
**Education**			<0.001			<0.001
Lower educated	61.5%	56.6%		60.2%	54.3%	
Higher educated	38.5%	43.4%		39.8%	45.7%	
**Employment**			<0.001			<0.001
Unemployed	46.5%	48.9%		45.5%	49.2%	
Employed	53.5%	51.1%		54.5%	50.8%	
**Longstanding illness**			<0.001			<0.001
No	54.5%	49.8%		54.5%	49.5%	
Yes	45.5%	50.2%		45.5%	50.5%	
**Current smoking**			<0.001			0.034
Non-smoker	83.2%	81.3%		84.2%	83.2%	
Current smoker	16.8%	18.7%		15.8%	16.8%	
**Current drinking**			0.54			0.88
No	24.0%	23.7%		24.0%	24.1%	
Yes	76.0%	76.3%		76.0%	75.9%	
**Household income**			0.45			<0.001
Top quintile	20.3%	21.1%		19.4%	21.5%	
2nd	21.0%	20.8%		21.9%	21.8%	
3rd	19.5%	19.9%		19.4%	19.9%	
4th	19.4%	19.4%		20.1%	19.2%	
Bottom quintile	19.8%	18.9%		19.2%	17.7%	
**Area deprivation**			<0.001			<0.001
1st - most deprived	20.2%	16.5%		19.6%	16.6%	
2nd	19.3%	18.4%		20.3%	20.3%	
3rd	20.1%	22.6%		20.2%	20.9%	
4th	20.2%	21.9%		20.2%	21.7%	
5th - least deprived	20.2%	20.7%		19.7%	20.6%	

Table S4: Descriptive analysis of Scotland versus England, 2013-2019 (DID model 4)

	2013-2017			2018-2019		
	**England**	**Scotland**	**p**	**England**	**Scotland**	**p**
**N**	40,914	22,570		16,382	9,713	
**Sex**			0.39			0.051
Female	55.6%	55.9%		55.2%	56.4%	
Male	44.4%	44.1%		44.8%	43.6%	
**Age groups**			<0.001			<0.001
16-19	4.2%	3.4%		4.2%	3.4%	
20-24	4.9%	5.2%		4.8%	4.2%	
25-34	14.3%	13.1%		13.7%	12.2%	
35-49	25.4%	23.3%		24.7%	22.4%	
50-59	16.7%	18.4%		17.4%	19.0%	
60+	34.5%	36.5%		35.2%	38.8%	
**Marital status**			<0.001			<0.001
Single/ Divorced/ Separated/ Widowed	35.6%	37.6%		35.2%	37.5%	
Married/ co-habited	64.4%	62.4%		64.8%	62.5%	
**Education**			<0.001			<0.001
Lower educated	62.8%	58.5%		60.2%	54.3%	
Higher educated	37.2%	41.5%		39.8%	45.7%	
**Employment**			<0.001			<0.001
Unemployed	46.3%	48.5%		45.5%	49.2%	
Employed	53.7%	51.5%		54.5%	50.8%	
**Longstanding illness**			<0.001			<0.001
No	56.3%	50.7%		54.5%	49.5%	
Yes	43.7%	49.3%		45.5%	50.5%	
**Current smoking**			<0.001			0.034
Non-smoker	82.3%	79.8%		84.2%	83.2%	
Current smoker	17.7%	20.2%		15.8%	16.8%	
**Current drinking**			0.89			0.88
No	23.5%	23.6%		24.0%	24.1%	
Yes	76.5%	76.4%		76.0%	75.9%	
**Household income**			0.2			<0.001
Top quintile	21.5%	21.0%		19.4%	21.5%	
2nd	21.2%	20.9%		21.9%	21.8%	
3rd	20.0%	20.3%		19.4%	19.9%	
4th	18.5%	19.3%		20.1%	19.2%	
Bottom quintile	18.7%	18.5%		19.2%	17.7%	
**Area deprivation**			<0.001			<0.001
1st - most deprived	19.5%	16.8%		19.6%	16.6%	
2nd	19.1%	19.7%		20.3%	20.3%	
3rd	20.3%	22.4%		20.2%	20.9%	
4th	20.4%	22.0%		20.2%	21.7%	
5th - least deprived	20.8%	19.1%		19.7%	20.6%	
