# Supplementary material for: An investigation into patterns of Alcohol drinking in Scotland after the introduction of minimum unit pricing
Source: PLoS One. 2024 Aug 1;19(8):e0308218. doi: 10.1371/journal.pone.0308218 (PMC11293661; doi:10.1371/journal.pone.0308218)
Supplement: S3 File — (PDF) [file pone.0308218.s003.pdf]

Supplementary 3: Preliminary investigation of the time trend of drinking rate in Scotland

The preliminary analysis was performed by using joinpoint regression to examine the time-trend of current drinking rate and also the drinking amount in Scotland from 2008-2021. Joinpoint regression, also known as a piecewise regression or segmented regression, allows researchers to interpret changes more accurately over time and to determine if those changes are statistically significant [1]. This technique has been extensively used in numerous epidemiology studies to identify and evaluate when/if changes in population parameters occur following interventions [1]. The joinpoint regression model can be written as:

$$y_i = \alpha + \beta_1 x_i + \delta_1 (x_i - \tau_1)^+ + \cdots + \delta_k (x_i - \tau_k)^+ + \varepsilon_i^{(k)}$$

$$\text{with } (x_i - \tau_k)^+ = x_i - \tau_k \text{ if } x_i > \tau_k, \text{ otherwise } (x_i - \tau_k)^+ = 0$$

where x is the time variable in ascending order from x1 to xn, y is the outcome variable varied in correspondence of time and $\tau_1, \ldots, \tau_k$ indicates the join points ($\tau_1 < \ldots < \tau_k$).

The model started from a minimum joinpoint (no joinpoint, assuming a straight line) to test for a linear trend throughout 2008-2021. Then, the model was expanded with one or up to three joinpoints to check if the added joinpoints are statistically significant in explaining the change of crude smoking rate. The Monte-Carlo permutation test is used to determine the number of joinpoints that gave the best model for explaining the time-trend of interested outcome (straight or multiple-segmented line) [2]. The final joinpoint model is selected using AIC and BIC to determine the best cutting point where a significant change in the intercept and slope between two segments of time presented.

Below are the results of joinpoint regression on two outcomes: current drinking rate and drinking amount in Scotland from 2008-2021.

- Current drinking rate: **Figure S*1*** below showed that the time trend of current drinking rate in Scotland had at least one significant breaking point, divided the drinking pattern into 2 periods: 2008-2012 and 2013-2021. Of these, the decline in earlier period was larger and significant at $p<0.05$ while the decline in drinking rate in 2013-2021 was not significant at p-0.05.
- Drinking amount: **Figure S*2*** showed the time trend of average weekly drinking amount in Scotland which also showed a significant breaking point in year 2013 as seen in current drinking rate.

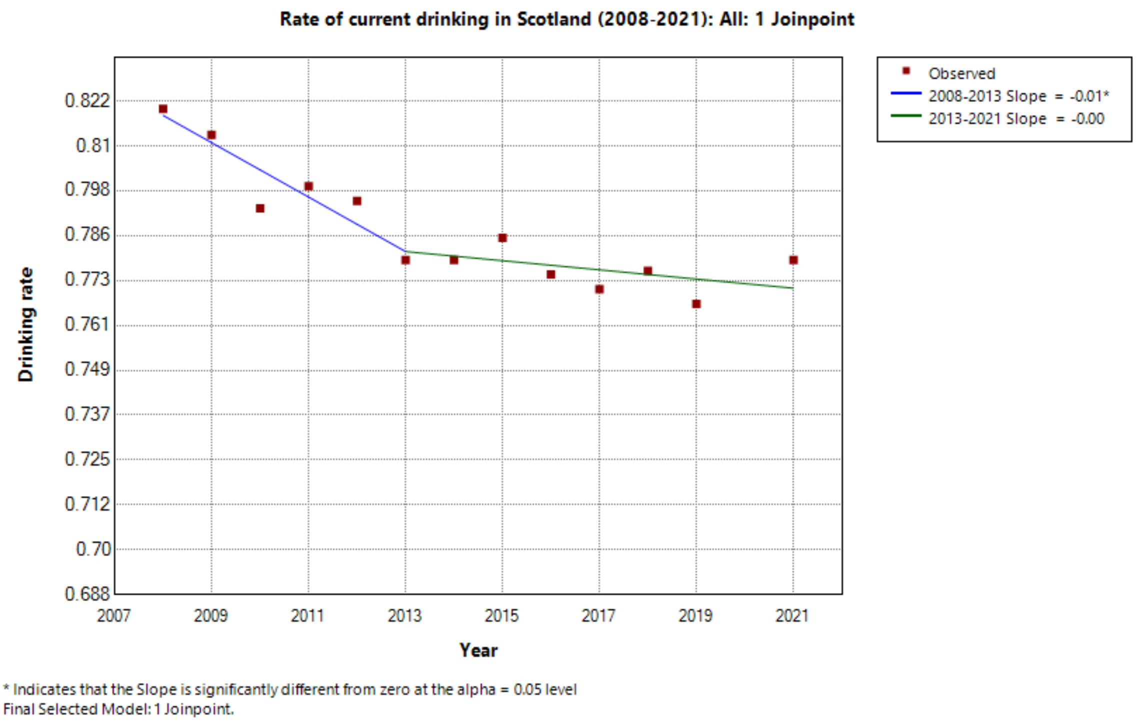

Figure S1: Time trend of current drinking rate in Scotland from 2008-2021

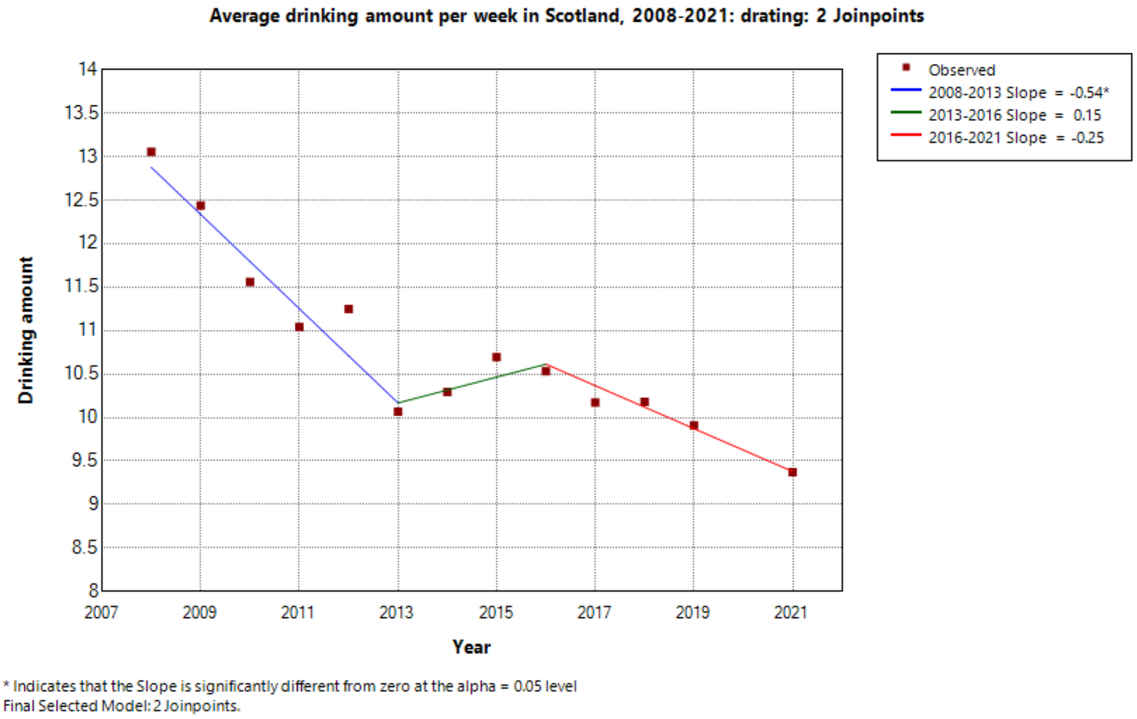

Figure S2: Time trend of drinking amount in Scotland from 2008-2021

1. Gillis D, Edwards BPM: **The utility of joinpoint regression for estimating population parameters given changes in population structure**. *Heliyon* 2019, **5**(11):e02515.
2. Kim HJ, Fay MP, Feuer EJ, Midthune DN: **Permutation tests for joinpoint regression with applications to cancer rates**. *Stat Med* 2000, **19**(3):335-351.
