# Supplementary material for: An investigation into patterns of Alcohol drinking in Scotland after the introduction of minimum unit pricing
Source: PLoS One. 2024 Aug 1;19(8):e0308218. doi: 10.1371/journal.pone.0308218 (PMC11293661; doi:10.1371/journal.pone.0308218)
Supplement: S4 File — (PDF) [file pone.0308218.s004.pdf]

Supplementary 4: Additional examination of drinking pattern in Scotland from 2008-2019 (excluding data post-COVID)

Table S1: Generalized ordered logistic models for drinking categories in Scotland, 2008-2019

Drinking category	Current drinking [a]	Heavy drinking [b]	Harmful drinking [c]
**Period**			
2008-2012	ref	ref	ref
2013-2017	0.87 (0.02) ***	0.87 (0.02) ***	0.87 (0.02) ***
2018-2019	0.87 (0.03) ***	0.87 (0.03) ***	0.87 (0.03) ***
**Male**			
**Age**	1.32 (0.04) ***	1.43 (0.04) ***	1.55 (0.09) ***
**Ethnic (White)**	0.99 (0.00) ***	1.00 (0.00)	1.00 (0.00) **
**Married/ cohabited**	8.09 (0.69) ***	4.02 (0.52) ***	4.85 (1.33) ***
**High education**	1.23 (0.04) ***	0.97 (0.03)	0.83 (0.05) **
**Employed**	1.43 (0.05) ***	1.15 (0.04) ***	0.93 (0.06)
**Current smoking**	1.74 (0.07) ***	1.06 (0.04) *	0.84 (0.05) **
**Long term illness**	1.17 (0.05) ***	1.94 (0.07) ***	2.65 (0.16) ***
**Equivalized income**	0.66 (0.02) ***	0.91 (0.03) **	1.19 (0.07) **
Top quintile			
2nd	ref	ref	ref
3rd	0.82 (0.03) ***	0.82 (0.03) ***	0.82 (0.03) ***
4th	0.67 (0.03) ***	0.67 (0.03) ***	0.67 (0.03) ***
Bottom quintile	0.54 (0.02) ***	0.54 (0.02) ***	0.54 (0.02) ***
**Area deprivation**	0.47 (0.02) ***	0.52 (0.03) ***	0.75 (0.06) **
1st - Most deprived			
2nd	ref	ref	ref
3rd	1.08 (0.04) **	1.08 (0.04) **	1.08 (0.04) **
4th	1.39 (0.07) ***	1.19 (0.06) ***	1.14 (0.10)
5th – Least deprived	1.36 (0.08) ***	1.13 (0.05) **	1.06 (0.10)

*N=49,404. *** p<0.001; ** p<0.05, *p<0.10. Odds ratios (SE) were weighted for complex survey design.*

a - Current drinking: abstainer (ref) vs. moderate/ hazardous/ harmful drinking.

b - Heavy drinking: abstainer / moderate (ref) vs. hazardous/ harmful drinking.

c - Harmful drinking: abstainer / moderate/ hazardous (ref) vs. harmful drinking.

Table S2: Negative binomial models on the weekly drinking amount in Scotland, 2008-2019

Alcohol consumption	Overall [a]	Moderate drinkers [b]	Hazardous drinkers [c]	Harmful drinkers [d]
**Period**				
2008-2012	ref	ref	ref	ref
2013-2017	0.93 (0.02)***	-0.05 (0.01)**	-0.01 (0.01)	-0.02 (0.03)
2018-2019	0.91 (0.03)**	-0.09 (0.02)***	0.00 (0.01)	-0.01 (0.04)
**Male**	2.00 (0.03)***	0.58 (0.01)***	0.39 (0.01)***	0.41 (0.03)***
**Age**	1.00 (0.00)***	0.00 (0.00)	0.00 (0.00)**	0.00 (0.00)**
**Ethnic (White)**	2.85 (0.22)***	0.40 (0.06)***	-0.01 (0.04)	0.09 (0.11)
**Married/ cohabited**	1.00 (0.02)	0.05 (0.01)**	0.00 (0.01)	-0.06 (0.03)*
**High education**	1.10 (0.02)***	0.08 (0.01)***	0.00 (0.01)	-0.03 (0.03)
**Employed**	1.02 (0.02)	0.08 (0.02)***	0.00 (0.01)	-0.08 (0.03)**
**Current smoking**	1.66 (0.04)***	0.11 (0.02)***	0.05 (0.01)***	0.11 (0.03)**
**Long term illness**	0.96 (0.02)**	-0.14 (0.01)***	0.01 (0.01)	0.09 (0.03)***
**Equivalized income**				
Top quintile	ref	ref	ref	ref
2nd	0.90 (0.02)***	-0.08 (0.02)***	0.00 (0.01)	-0.04 (0.05)
3rd	0.80 (0.02)***	-0.15 (0.02)***	0.01 (0.01)	-0.01 (0.05)
4th	0.67 (0.02)***	-0.22 (0.02)***	-0.01 (0.01)	-0.05 (0.06)
Bottom quintile	0.71 (0.03)***	-0.25 (0.03)***	0.01 (0.01)	0.12 (0.07)*
**Area deprivation**				
1st - Most deprived	ref	ref	ref	ref
2nd	0.99 (0.03)	0.00 (0.02)	0.00 (0.01)	-0.08 (0.04)**
3rd	1.09 (0.04)**	0.03 (0.02)	0.00 (0.01)	-0.02 (0.05)
4th	1.07 (0.03)**	0.07 (0.02)**	0.01 (0.01)	-0.10 (0.04)**
5th – Least deprived	1.15 (0.04)***	0.11 (0.02)***	0.00 (0.01)	-0.06 (0.04)
**Margins of drinking amount (UK standard units)**				
**Period**				
**2008-2012**	10.40 (0.14)	5.54 (0.05)	26.26 (0.13)	70.07 (1.43)
**2013-2017**	9.64 (0.15)	5.29 (0.06)	26.09 (0.15)	68.73 (1.28)
**2018-2019**	9.46 (0.24)	5.06 (0.09)	26.28 (0.26)	69.65 (2.56)

a Overall N = 49,404; b Moderate drinkers = 32,089; c Hazardous drinkers = 8,049; d Harmful drinkers = 1,884.

***** p<0.001; ** p<0.05; * p<0.10. Coefficients (SE) were weighted for complex survey designs. Margins in the three bottom rows were estimated from negative binomial models.**
